# Rapid Self-Alignment Method for Fixed-Wing Aircraft Aided by Runway Heading

**DOI:** 10.3390/s26154839

**Published:** 2026-07-31

**Authors:** Rui Li, Qiangwen Fu, Bingbing Gao, Qi Zhou, Qi Xue, Lihang Cui

**Affiliations:** 1 School of Automation, Northwestern Polytechnical University, Xi’an 710129, China; lirio@mail.nwpu.edu.cn (R.L.); qi_xue@mail.nwpu.edu.cn (Q.X.); 2 Shenzhen Research Institute, Northwestern Polytechnical University, Shenzhen 518057, China; 3 Xi’an Flight Control Research Institute, Aviation Industry Corporation of China, Xi’an 710065, China; zhouqis@139.com; 4 Hubei Key Laboratory of Aerospace Intelligent Control Technology, Hubei Sanjiang Aerospace Hongfeng Control Co., Ltd., Xiaogan 432000, China; clhcyf@163.com

**Keywords:** in-motion alignment, in-flight alignment, inertial navigation system, initial alignment, Kalman filter

## Abstract

**Highlights:**

**What are the main findings?**

- A runway-heading-constrained self-alignment method for SINS during runway taxiing is proposed, with no requirement for external aiding sensors.
- A 15-state Kalman filter incorporating combined virtual velocity and displacement constraints is developed to significantly accelerate azimuth alignment convergence.

**What are the implications of the main findings?**

- The proposed approach significantly reduces the initial alignment time, thus improving the autonomy and rapid-response capability of aircraft SINS.
- This runway-constrained strategy provides a practical, easy-to-implement rapid alignment solution for both military and civil aviation applications.

**Abstract:**

To improve the speed and accuracy of airborne inertial navigation systems during ground initial alignment, this paper proposes a rapid self-alignment method suitable for fixed-wing aircraft during the taxi-to-takeoff phase on the runway. This approach leverages pre-surveyed runway heading data to transform the velocity vector calculated in the navigation frame to the runway frame. By utilizing the geometric constraint that the aircraft’s actual position during ground taxiing must remain within the runway boundaries and closely follow the runway centerline, the proposed method employs a dedicated Kalman filter to achieve rapid initial alignment. Both simulations and flight tests demonstrate that the proposed method can achieve rapid initial alignment within 30 s during the runway taxiing phase, with attitude, velocity, and position accuracy levels comparable to those of GNSS-aided in-motion alignment. This method eliminates the need for external aiding equipment and can serve as an emergency initial alignment solution for airborne inertial navigation systems under GNSS outage conditions, effectively enhancing the aircraft’s autonomy and rapid response capability.

## 1. Introduction

Inertial navigation systems (INS) are characterized by inherent advantages of full autonomy, high concealment, and complete navigation information, making them indispensable navigation equipment for critical aircraft [1,2]. Prior to performing navigation tasks, the INS must complete initial alignment, during which alignment accuracy and alignment speed are the two key metrics [3,4,5]. On the one hand, initial alignment accuracy largely determines the subsequent navigation precision of the INS and further influences flight safety, operational effectiveness, and mission execution capability [6,7,8]. On the other hand, alignment speed directly impacts aircraft preparation time, as an excessively long INS alignment process will impose a critical constraint on the aircraft’s rapid-response capability [9].

Under stationary base conditions, the performance of initial alignment is fundamentally limited by the precision of inertial sensors due to inherent observability constraints. Specifically, the horizontal accelerometer bias determines the horizontal attitude alignment accuracy; the east gyroscope drift governs the azimuth alignment accuracy, and gyroscope angle random walk is one of the primary factors affecting the convergence time of azimuth alignment [10,11]. Research on SINS initial alignment has a long history. Early foundational research, including Lipton’s work on moving base alignment [12], laid the theoretical groundwork for alignment problem formulation. Comprehensive overviews and the fundamental analytical frameworks of SINS alignment can also be found in the classic literature by Titterton and Weston [13]. Over the decades, various advanced initial alignment methods have been developed, including conventional analytical alignment [14], parameter identification alignment [15], Kalman filter alignment [10,16], inertial-frame alignment [17,18,19], and Lie group theory-based alignment [20]. While these methods differ in aspects of information utilization, disturbance rejection, noise suppression capability, convergence speed, and algorithmic complexity, their ultimate alignment accuracy under stationary base conditions remains consistent. Due to sensor noise and external disturbances, INS initial alignment typically requires 5–8 min of stationary time to achieve the ultimate alignment precision, which significantly limits the maneuverability and rapid response capability of military combat aircraft.

In-flight alignment (IFA) technology enables the INS to rapidly complete initial alignment after an aircraft’s emergency takeoff, typically with the aid of external navigation systems such as the global navigation satellite system (GNSS) [21,22], celestial navigation system (CNS) [23,24], or airborne radar systems [25]. By leveraging external attitude, velocity, or position information and enhancing system observability through aircraft maneuvers, IFA can achieve higher alignment accuracy within a shorter time.

The fundamental interaction mechanisms and calibration algorithms between the aligned INS and external reference systems on a moving base have been extensively studied by researchers such as Vodicheva [26] to optimize this data fusion process. However, reliance on external equipment restricts its operational scenarios and compromises the autonomy of the INS. Moreover, the alignment accuracy is highly sensitive to the intensity of the aircraft maneuvers and the measurement errors of the aiding systems. Additionally, performing maneuvers immediately after takeoff, when the INS has not yet achieved full navigation capability, increases pilot workload and may compromise flight safety.

To address the challenge of rapid ground initial alignment, a high-precision optical aiding system for heading measurement was developed in [27]. After pre-calibration, this system is capable of determining the heading of the airborne INS within three minutes by measuring designated markers on the aircraft; however, it remains a non-autonomous approach that relies on external equipment. Beyond purely optical auxiliary systems, vision-based data fusion has also been introduced to exploit features of the runway environment. For instance, Grof et al. proposed an IMU-camera data fusion scheme focused on runway contour recognition to estimate and reduce aircraft orientation errors during the runway-relative positioning process [28]. In [29], the initial alignment for carrier-based aircraft was accomplished during the high-acceleration catapult launch phase by the fusion of runway heading information from the shipboard master INS and auxiliary velocity measurements from laser Doppler velocimetry (LDV).

Building upon prior research on autonomous INS alignment [10,11,15], this paper investigates the rapid self-alignment problem of fixed-wing aircraft during the runway taxiing phase. Inspired by the non-holonomic constraint (NHC)-assisted alignment strategy for land vehicles in [30], this paper establishes a dedicated alignment framework under the runway coordinate frame using the pre-surveyed runway heading. In conventional NHC-based methods, the accurate heading of the vehicle remains unknown, and consequently, the lateral and vertical velocity constraints fail to effectively improve heading estimation performance. However, the method proposed in this paper, formulated using velocity and displacement constraints within the runway coordinate frame, enables the taxiing acceleration to directly excite the heading error dynamics, thereby achieving faster convergence and higher estimation accuracy.

## 2. Runway-Constrained Alignment Algorithm

### 2.1. Principle of Runway Velocity Constraints

The coordinate frames adopted throughout this study are formally defined as follows:

- Body Frame (b-frame): The $x_{b}$, $y_{b}$ and $z_{b}$ axes of the body frame are oriented according to the Right–Forward–Up (RFU) convention.
- Navigation Frame (n-frame): The $x_{n}$, $y_{n}$ and $z_{n}$ axes of the navigation frame are aligned with the local geographic East-North-Up (ENU) directions.
- Runway Frame (p-frame): The $x_{p}$-axis is perpendicular to the runway pointing to the right; the $y_{p}$-axis is aligned with the runway centerline pointing forward; and the $z_{p}$-axis points vertically upward.

**Figure 1:**
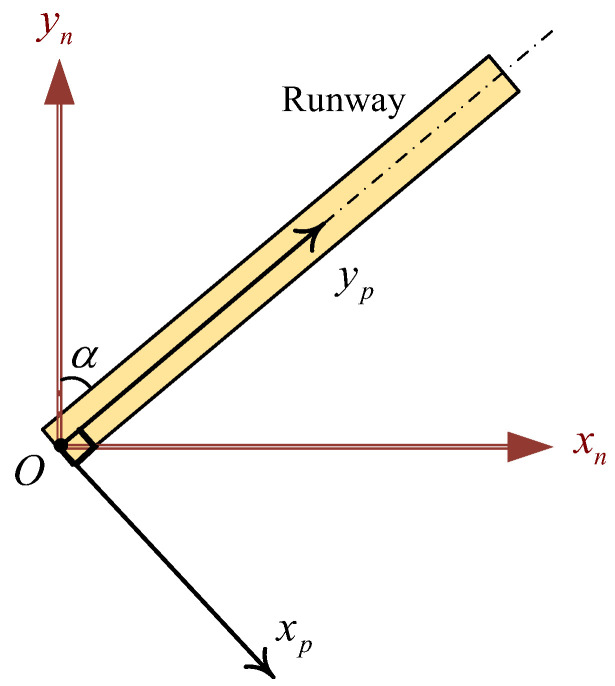
Relationship between the navigation frame and the runway frame.

The relationship between the navigation frame and the runway frame is illustrated in Figure 1.

In Figure 1, the angle between the $y_{p}$-axis of the runway frame and the $y_{n}$-axis of the navigation frame is defined as the pre-surveyed runway heading angle (α), which is treated as known information throughout this study. The coordinate transformation matrix from the n-frame to the p-frame is denoted as $C_{n}^{p}$, which can be expressed as follows:

(1) $$C_{n}^{p}=\left[\begin{array}{ccc} \cos\alpha & -\sin\alpha & 0\\ \sin\alpha & \cos\alpha & 0\\ 0 & 0 & 1 \end{array}\right]$$

The velocity resolved in the n-frame can be transformed into the p-frame via the following transformation relation:

(2) $$v^{p}=C_{n}^{p}v^{n}$$

where $v^{n}$ and $v^{p}$ denote the velocity vectors in the navigation and runway frames, respectively.

An installation misalignment exists between the IMU sensor frame and the aircraft body frame, and the aircraft heading may dynamically adjust relative to the runway during taxiing. Therefore, if the runway heading angle is directly assigned to the INS, there will inevitably be significant errors.

However, similar to ground vehicles, an aircraft taxiing on a runway is subject to inherent kinematic constraints [31,32]. Although a considerable and dynamically varying misalignment angle exists between the body frame and the runway frame, the aircraft is required to maintain its velocity vector aligned with the runway centerline during the taxiing phase to guarantee a safe takeoff. Consequently, while the longitudinal velocity component along the runway remains unknown during the taxiing phase, the velocity components along the lateral $x_{p}$-axis and vertical $z_{p}$-axis can be approximated as zero in the absence of sideslip or jumping. Thus, two virtual velocity measurements along the lateral and vertical directions can be obtained.

(3) $$\left\{\begin{array}{l} v_{x}^{p}=0\\ v_{z}^{p}=0 \end{array}\right.$$

In practice, the corresponding virtual-measurement errors $\delta v_{x}^{p}$ and $\delta v_{z}^{p}$ can be modeled as zero-mean white noise, with their variances adjusted to reflect the degree of constraint violation during taxiing. Compared with the full three-dimensional velocity-aided filter update, Equation (3) only provides two-dimensional velocity observations along the lateral and vertical axes of the runway frame, and is therefore referred to as a non-holonomic constraint.

However, this constraint differs fundamentally from the NHC commonly applied to land vehicles. During the taxiing phase, the aircraft’s motion is confined to a straight-line trajectory along a runway whose heading has been accurately surveyed in advance. Consequently, the velocity observation vector is obtained using the precisely known transformation matrix $C_{n}^{p}$, rather than the INS-computed attitude matrix $C_{n}^{b}$ that inherently contains errors. Furthermore, although the constraint in Equation (3) may not hold strictly at every instant, overall, the aircraft generally travels along the runway centerline without deviating beyond the runway boundaries; thus, the displacement constraint inherently exists.

### 2.2. State-Space Model

#### 2.2.1. Error-State Model

To keep the state dimension consistent during the alignment and subsequent navigation phases, and to avoid reinitializing the filter, a classical 15-dimensional error-state vector consistent with the INS/GNSS integrated navigation framework is adopted. The state vector is defined as follows:

(4) $$X(t)={\left[\begin{array}{ccccc} {\left(\phi^{n}\right)}^{T} & {\left(\delta v^{n}\right)}^{T} & {\left(\delta p\right)}^{T} & {\left(\varepsilon^{b}\right)}^{T} & {\left(\nabla^{b}\right)}^{T} \end{array}\right]}^{T}$$

where X(t) is a 15-dimensional state vector; $\phi^{n}=\left[\begin{array}{ccc} \phi_{E} & \phi_{N} & \phi_{U} \end{array}\right]$, $\delta v^{n}=\left[\begin{array}{ccc} \delta v_{E} & \delta v_{N} & \delta v_{U} \end{array}\right]$ and $\delta p=\left[\begin{array}{ccc} \delta \lambda & \delta L & \delta h \end{array}\right]$ denote the attitude misalignment angles, velocity errors, and position errors, respectively; $\varepsilon^{b}$ and $\nabla^{b}$ represent the constant gyro drifts and constant accelerometer biases, respectively.

The error propagation equations of the strapdown inertial navigation system (SINS) are given as follows [33]:

(5) $${\dot{\phi }}^{n}=-\omega_{in}^{n}\times \phi^{n}+M_{1}\delta v^{n}+M_{2}\delta p-C_{b}^{n}\varepsilon^{b}-\varepsilon_{w}^{n}$$

(6) $$\delta {\dot{v}}^{n}=f^{n}\times \phi^{n}+M_{3}\delta v^{n}+M_{4}\delta p+C_{b}^{n}\nabla^{b}+\nabla_{w}^{n}$$

(7) $$\delta \dot{p}=M_{5}\delta v^{n}+M_{6}\delta p$$

(8) $${\dot{\varepsilon }}^{b}=0_{3\times 1}$$

(9) $${\dot{\nabla }}^{b}=0_{3\times 1}$$

(10) $$M_{1}=\left[\begin{array}{ccc} 0 & -1/\left(R_{M}+h\right) & 0\\ 1/\left(R_{N}+h\right) & 0 & 0\\ \tan L/\left(R_{N}+h\right) & 0 & 0 \end{array}\right]$$

(11) $$M^{\prime }=\left[\begin{array}{ccc} 0 & 0 & 0\\ -\omega_{ie}\sin L & 0 & 0\\ \omega_{ie}\cos L & 0 & 0 \end{array}\right],\;M\prime \prime =\left[\begin{array}{ccc} 0 & 0 & \frac{v_{N}}{{\left(R_{M}+h\right)}^{2}}\\ 0 & 0 & -\frac{v_{E}}{{\left(R_{N}+h\right)}^{2}}\\ \frac{v_{E}\sec^{2}L}{R_{N}+h} & 0 & -\frac{v_{E}\tan L}{{\left(R_{N}+h\right)}^{2}} \end{array}\right]$$

(12) $$M_{2}=M^{\prime }+M^{\prime \prime }$$

(13) $$M_{3}=\left(v^{n}\times \right)M_{1}-\left[\left(2\omega_{ie}^{n}+\omega_{en}^{n}\right)\times \right]$$

(14) $$M_{4}=\left(v^{n}\times \right)\left(2M^{\prime }+M^{\prime \prime }\right)$$

(15) $$M_{5}=\left[\begin{array}{ccc} 0 & \frac{1}{R_{M}+h} & 0\\ \frac{\sec L}{R_{N}+h} & 0 & 0\\ 0 & 0 & 1 \end{array}\right],\;M_{6}=\left[\begin{array}{ccc} 0 & 0 & -\frac{v_{N}}{{\left(R_{M}+h\right)}^{2}}\\ \frac{v_{E}\sec L\tan L}{R_{N}+h} & 0 & -\frac{v_{E}\sec L}{{\left(R_{N}+h\right)}^{2}}\\ 0 & 0 & 0 \end{array}\right]$$

where $\omega_{ie}$ is the Earth’s rotation rate; $\omega_{en}^{n}$ is the angular rate of the n-frame with respect to the Earth frame; $\omega_{in}^{n}$ is the angular rate of the n-frame with respect to the inertial frame; $C_{b}^{n}$ is the attitude matrix from the b-frame to the navigation frame; $\varepsilon_{w}^{n}=C_{b}^{n}\varepsilon_{w}^{b}(t)$ and $\nabla_{w}^{n}=C_{b}^{n}\nabla_{w}^{b}(t)$ denote the equivalent gyroscope and accelerometer process noise, respectively; L and h denote the geographic latitude and altitude, respectively; $v_{E}$ and $v_{N}$ denote the eastward and northward velocity components, respectively; $R_{M}$ and $R_{N}$ denote the meridian and prime-vertical radii of curvature, respectively. The gyroscope drifts and accelerometer biases are modeled as constant error states to be estimated.

Based on the SINS error model described above, the corresponding continuous-time state-space equation can be formulated as follows:

(16) $$\dot{X}(t)=F(t)X(t)+G(t)W(t)$$

where F(t) and G(t) are deterministic time-varying matrices with respect to the parameter t, while W(t) denotes a continuous-time zero-mean white noise vector.

(17) $$F(t)=\left[\begin{array}{ccccc} -\omega_{in}^{n}\times & M_{1} & M_{2} & -C_{b}^{n} & 0_{3\times 3}\\ f^{n}\times & M_{3} & M_{4} & 0_{3\times 3} & C_{b}^{n}\\ 0_{3\times 3} & M_{5} & M_{6} & 0_{3\times 3} & 0_{3\times 3}\\ 0_{3\times 3} & 0_{3\times 3} & 0_{3\times 3} & 0_{3\times 3} & 0_{3\times 3}\\ 0_{3\times 3} & 0_{3\times 3} & 0_{3\times 3} & 0_{3\times 3} & 0_{3\times 3} \end{array}\right]$$

(18) $$G(t)=\left[\begin{array}{cc} -C_{b}^{n} & 0_{3\times 3}\\ 0_{3\times 3} & C_{b}^{n}\\ 0_{9\times 3} & 0_{9\times 3} \end{array}\right]$$

(19) $$W(t)=\left[\begin{array}{c} \varepsilon_{w}^{b}(t)\\ \nabla_{w}^{b}(t) \end{array}\right]$$

In Equation (19), $\varepsilon_{w}^{b}(t)$ refers to the gyroscope angular random walk (ARW) white noise process with a power spectral density (PSD) of $q_{g}$, while $\nabla_{w}^{b}(t)$ corresponds to the accelerometer velocity random walk (VRW) white noise process with a PSD of $q_{a}$. The vector W(t) satisfies the following statistical characteristics

(20) $$E\left[W(t)\right]=0,\;\;E\left[W(t)W^{T}(\tau )\right]=\left[\begin{array}{cc} q_{g}I_{3} & 0_{3\times 3}\\ 0_{3\times 3} & q_{a}I_{3} \end{array}\right]\delta (t-\tau )$$

where $E\left[\cdot \right]$ represents the mathematical expectation operator, and $\delta \left(t\right)$ is the Dirac delta function.

If $\sigma_{g}$ is the standard deviation of the noise on the gyro angular-rate measurements and $\sigma_{a}$ is the standard deviation of the noise on the accelerometer specific-force measurements, then the PSDs of the gyro and accelerometer noise are [34]

(21) $$q_{g}=\sigma_{g}^{2}T_{i},\;q_{a}=\sigma_{a}^{2}T_{i}$$

where $T_{i}$ is the sample interval.

According to Equation (21), $\sqrt{q_{g}}$ is commonly defined as the gyroscope ARW coefficient with units of ${}^{\circ}/\sqrt{h}$ or $({}^{\circ}/h)/\sqrt{\mathrm{Hz}}$, while $\sqrt{q_{a}}$ denotes the accelerometer VRW coefficient with units of $\left(m/s\right)/\sqrt{h}$, $\mu g\cdot \sqrt{s}$ or $\mu g/\sqrt{\mathrm{Hz}}$ ($1\;\mu g=9.8\times 10^{-6}\;m/s^{2}$).

Based on the theory for solving linear stochastic differential equations, the discretized form of the continuous-time state Equation (16) can be obtained

(22) $$X_{k}=\Phi_{k,k-1}X_{k-1}+W_{k-1}$$

where $\Phi_{k,k-1}$ is the discrete-time state transition matrix from time $t_{k-1}$ to $t_{k}$, and $W_{k-1}$ is the discrete process noise.

Since the sampling interval is sufficiently small, the first-order approximation is adopted to discretize the continuous-time system matrix, yielding

(23) $$\Phi_{k,k-1}=e^{\int_{t_{k-1}}^{t_{k}}F\left(t\right)dt}\approx I+F\left(t_{k}\right)T_{s}$$

where $F\left(t_{k}\right)$ is the continuous-time system matrix evaluated at time epoch $t_{k}$, and $T_{s}=t_{k}-t_{k-1}$ denotes the state propagation interval, which is set to 0.1 s in this paper.

Considering the small propagation interval and the orthogonality property of the attitude matrix $C_{b}^{n}$, it can be derived that

(24) $$E\left[W_{k}\right]=0,\;E[W_{k}W_{j}^{T}]=Q_{k}\delta (k-j)$$

where

(25) $$Q_{k}\approx Q_{t}T_{s}=\left[\begin{array}{ccc} q_{g}I_{3} & 0_{3\times 3} & 0_{3\times 6}\\ 0_{3\times 3} & q_{a}I_{3} & 0_{3\times 6}\\ 0_{6\times 3} & 0_{6\times 3} & 0_{6\times 6} \end{array}\right]$$

in which the detailed parameter configuration for the process noise covariance matrix $Q_{t}$ is specified in Appendix A.

#### 2.2.2. Velocity-Constrained Alignment Model

During the taxiing phase, the velocity error projected onto the runway frame can be obtained from Equation (2) as follows:

(26) $$\delta v^{p}=C_{n}^{p}\delta v^{n}$$

Based on the constraint conditions in Equations (3) and (26), the measurement equation for the runway-frame velocity constraint is formulated as follows:

(27) $$Z_{v}(t)=\left[\begin{array}{c} v_{x}^{p}\\ v_{z}^{p} \end{array}\right]=\left[\begin{array}{ccccc} 0_{2\times 3} & C_{n(1,3)}^{p} & 0_{2\times 3} & 0_{2\times 3} & 0_{2\times 3} \end{array}\right]X(t)+V_{v}(t)$$

where $C_{n(1,3)}^{p}$ is the observation matrix obtained by selecting the first and third rows of the matrix $C_{n}^{p}$.

(28) $$C_{n(1,3)}^{p}=\left[\begin{array}{ccc} \cos\alpha & -\sin\alpha & 0\\ 0 & 0 & 1 \end{array}\right]$$

In Equation (27), $V_{v}(t)={\left[\begin{array}{cc} V_{vx}(t) & V_{vz}(t) \end{array}\right]}^{T}$ denotes the disturbance velocities in the $x_{p}$ and $z_{p}$ axes induced by factors including runway heading error, crosswind, sideslip, uneven runway pavement, and aircraft structural vibration. Considering the inherent difficulty in accurately modeling such disturbances, these disturbance velocities are uniformly characterized as zero-mean white noise processes with a PSD of $r_{v}$.

(29) $$E\left[V_{v}(t)\right]=0,\;E\left[V_{v}(t)V_{v}^{T}(\tau )\right]=r_{v}\delta \left(t-\tau \right)$$

The corresponding discretized measurement equation derived from Equation (27) is

(30) $$Z_{v,k}=\left[\begin{array}{c} v_{x,k}^{p}\\ v_{z,k}^{p} \end{array}\right]=H_{v,k}X_{k}+V_{v,k}$$

Specifically, $H_{v,k}$ denotes the discretized measurement matrix, and $V_{v,k}$ represents the discretized velocity measurement noise vector at time instant $t_{k}$.

(31) $$H_{v,k}=\left[\begin{array}{ccccc} 0_{2\times 3} & C_{n,k(1,3)}^{p} & 0_{2\times 3} & 0_{2\times 3} & 0_{2\times 3} \end{array}\right]$$

(32) $$E\left[V_{v,k}\right]=0,\;E\left[V_{v,k}V_{v,j}^{T}\right]=R_{v}\delta (k-j)$$

where $R_{v}=R_{v}/T_{m}$ denotes the velocity measurement noise variance that characterizes the degree of NHC violation during the taxiing phase, with its preset values provided in Appendix A. $T_{m}$ refers to the filtering update cycle, with a value of 0.1 s.

From the derived velocity measurement equation, it can be concluded that the SINS can achieve autonomous in-motion alignment not by relying on any external aiding equipment, but by using only the pre-surveyed runway heading data. This approach guarantees high system autonomy and strong robustness against external disturbances such as GNSS outages, radar interference, or the unavailability of celestial observations.

#### 2.2.3. Displacement-Constraint Alignment Model

During the taxiing phase, the aircraft may undergo minor orientation adjustments, which can induce instantaneous fluctuations in lateral velocity $v_{x}^{p}$ and consequently result in measurement errors. To mitigate this issue, a complementary displacement-constraint model can be constructed by integrating velocity. The underlying principle of the latter is that the aircraft generally taxis along a straight path near the runway centerline; therefore, the displacement components along the lateral $x_{p}$-axis and vertical $z_{p}$-axis can be approximated as zero.

The displacement vector in the runway frame is obtained by integrating the velocity.

(33) $$s^{p}=\int_{0}^{T}v^{p}dt=\int_{0}^{T}C_{n}^{p}v^{n}dt$$

The displacement components along the $x_{p}$-axis and $z_{p}$-axis are selected as the measurement values.

(34) $$Z_{s}=\left[\begin{array}{c} s_{x}^{p}\\ s_{z}^{p} \end{array}\right]$$

where $s_{x}^{p}$ and $s_{z}^{p}$ denote the lateral and vertical components of the displacement vector $s^{p}$, respectively.

The discretized displacement constraint measurement equation is directly formulated as follows:

(35) $$Z_{s,k}=\left[\begin{array}{c} s_{x,k}^{p}\\ s_{z,k}^{p} \end{array}\right]=H_{s,k}X_{k}+V_{s,k}$$

Accordingly, $H_{s,k}$ denotes the discretized measurement matrix, and $V_{s,k}$ represents the discretized displacement measurement noise vector.

(36) $$H_{s,k}=\left[\begin{array}{ccccc} 0_{2\times 3} & 0_{2\times 3} & M_{n,k(1,3)}^{p} & 0_{2\times 3} & 0_{2\times 3} \end{array}\right]$$

where $M_{n,k(1,3)}^{p}$ denotes the matrix formed by extracting the first and third rows of the matrix $M_{n,k}^{p}$ at time instant $t_{k}$, which is defined as follows:

(37) $$M_{n,k}^{p}=C_{n,k}^{p}\left[\begin{array}{ccc} (R_{N,k}+h_{k})\cos L_{k} & 0 & 0\\ 0 & R_{M,k}+h_{k} & 0\\ 0 & 0 & 1 \end{array}\right]$$

(38) $$M_{n,k(1,3)}^{p}=\left[\begin{array}{ccc} \cos\alpha (R_{N,k}+h_{k})\cos L & -\sin\alpha (R_{M,k}+h_{k}) & 0\\ 0 & 0 & 1 \end{array}\right]$$

In Equation (35), $V_{s,k}={\left[\begin{array}{cc} V_{sx,k} & V_{sz,k} \end{array}\right]}^{T}$ represents the disturbance displacements caused by multiple factors that drive the aircraft away from the runway centerline. These difficult-to-model disturbance displacements are treated as zero-mean white noise.

(39) $$E\left[V_{s,k}\right]=0,\;E\left[V_{s,k}V_{s,j}^{T}\right]=R_{s}\delta (k-j)$$

where $R_{s}$ denotes the displacement measurement noise variance, with its preset values specified in Appendix A.

As indicated by the displacement measurement equation, the alignment process employs the displacement errors in the runway frame, rather than the heading error between the INS and the runway, as measurements to estimate the INS attitude misalignment angles $\phi^{n}$. Compared with the velocity-constrained measurement model, the measurement noise covariance induced by interference can be more readily determined in the displacement-constrained measurement model. In practical applications, either Equation (27) or Equation (35) can be applied independently, or both can be employed simultaneously to enhance the observability of the system.

The primary distinction between the velocity-constrained and displacement-constrained measurement schemes lies in their measurement error sources. The former stems from lateral velocity perturbations during ground movement, whereas the latter concerns lateral displacement from the runway centerline during taxiing. Specifically, the maximum lateral velocity error arises when the aircraft is near the runway centerline, while the maximum displacement error occurs when the lateral disturbance velocity is approximately zero. Therefore, the two measurement schemes exhibit strong complementary characteristics.

Similar to integrated INS/GNSS navigation systems that jointly use position and velocity measurements, the velocity constraint in this method accelerates heading convergence, whereas the displacement constraint improves alignment stability and end-of-alignment positioning accuracy.

### 2.3. Workflow of Runway-Constrained Alignment

Based on the preceding analysis, the workflow of the proposed rapid self-alignment method for fixed-wing aircraft during the taxi-to-takeoff phase is outlined as follows:

(1) When the aircraft is parked at the runway threshold (as shown in Figure 1) with its nose aligned along the runway centerline, the SINS is initialized with the following initial conditions: the initial position is set to the runway origin coordinates; the initial velocity is set to zero; the initial pitch and roll angles are set to zero; and the initial heading angle is assigned the known runway heading angle α.
(2) Following initialization, the INS executes pure inertial navigation computation while simultaneously updating the state vector in accordance with Equation (22).
(3) Upon determining that the aircraft is in a stationary state prior to taxiing, a three-dimensional zero-velocity update (ZUPT) filter is employed.

(40) $$Z_{0,k}=v_{k}^{n}=H_{0}X_{k}+V_{0,k}$$

in which $H_{0}=\left[\begin{array}{ccccc} 0_{3\times 3} & I_{3\times 3} & 0_{3\times 3} & 0_{3\times 3} & 0_{3\times 3} \end{array}\right]$ is the measurement matrix; $V_{0,k}$ denotes the velocity white noise during the stationary phase, which satisfies the conditions $E\left[V_{0,k}\right]=0$ and $E\left[V_{0,k}V_{0,j}^{T}\right]=R_{v0}\delta (k-j)$, where $R_{v0}$ is the corresponding noise variance.

(4) During the taxiing phase, filter updates are executed in accordance with the velocity measurement model (30), and/or the displacement measurement model (35).
(5) Once the aircraft leaves the runway surface (e.g., as indicated by the weight-on-wheels signal becoming invalid), the initial alignment process is terminated.

### 2.4. Kalman Filter Design

Based on the discretized state equation, the state prediction is formulated as follows:

(41) $${\hat{X}}_{k/k-1}=\;\Phi_{k/k-1}{\hat{X}}_{k-1}\;$$

where ${\hat{X}}_{k/k-1}$ denotes the predicted state estimate, and its value ${\hat{X}}_{0}$ is provided in Appendix A.

The covariance prediction equation is [35]

(42) $$P_{k/k-1}=\Phi_{k/k-1}P_{k-1}\Phi_{k/k-1}^{T}+Q_{k-1}$$

where $P_{k/k-1}$ is the predicted error covariance matrix, and $Q_{k-1}=Q_{t}T_{s}$ denotes the process noise covariance matrix. The preset values of $P_{0}$ and $Q_{t}$ are listed in Appendix A.

The Kalman gain is computed as follows:

(43) $$K_{k}=P_{k/k-1}H_{k}^{T}{\left(H_{k}P_{k/k-1}H_{k}^{T}+R_{k}\right)}^{-1}$$

where $K_{k}$ is the Kalman gain, $H_{k}$ is the measurement matrix, and $R_{k}$ is the measurement-noise covariance matrix.

The state estimate is updated according to

(44) $${\hat{X}}_{k}={\hat{X}}_{k/k-1}+K_{k}\left(Z_{k}-H_{k}{\hat{X}}_{k/k-1}\right)$$

where $Z_{k}$ denotes the measurement vector and ${\hat{X}}_{k}$ represents the updated state estimate.

The covariance matrix is updated as follows:

(45) $$P_{k}=\left(I-K_{k}H_{k}\right)P_{k/k-1}$$

where $P_{k}$ is the updated error covariance matrix.

Equations (43)–(45) constitute the filtering update process. As the proposed method may involve up to three types of measurement updates, a sequential processing approach is employed for filter updating. During the ZUPT filtering phase, $Z_{k}$, $H_{k}$, and $R_{k}$ are substituted with $Z_{0,k}$, $H_{0}$, and $R_{v0}$ for measurement update; during velocity constraint filtering, $Z_{k}$, $H_{k}$, and $R_{k}$ are replaced by $Z_{v,k}$, $H_{v,k}$, and $R_{v}$; during displacement constraint filtering, $Z_{k}$, $H_{k}$, and $R_{k}$ are replaced by $Z_{s,k}$, $H_{s,k}$, and $R_{s}$.

Finally, the estimated attitude, velocity, and position errors are fed back into the navigation solution to compensate for accumulated navigation errors, thereby enhancing alignment performance.

The filter initialization, including the initial state vector, the initial error covariance matrix, and the process and measurement noise covariance matrices, is set according to the sensor specifications and experimental conditions. The detailed parameter settings are summarized in Appendix A. Unless otherwise specified, the same filter configuration is adopted for both simulation and flight-test evaluations to ensure a fair and consistent performance assessment of the proposed alignment method.

## 3. Simulation Verification

### 3.1. Simulation Conditions

**Figure 2:**
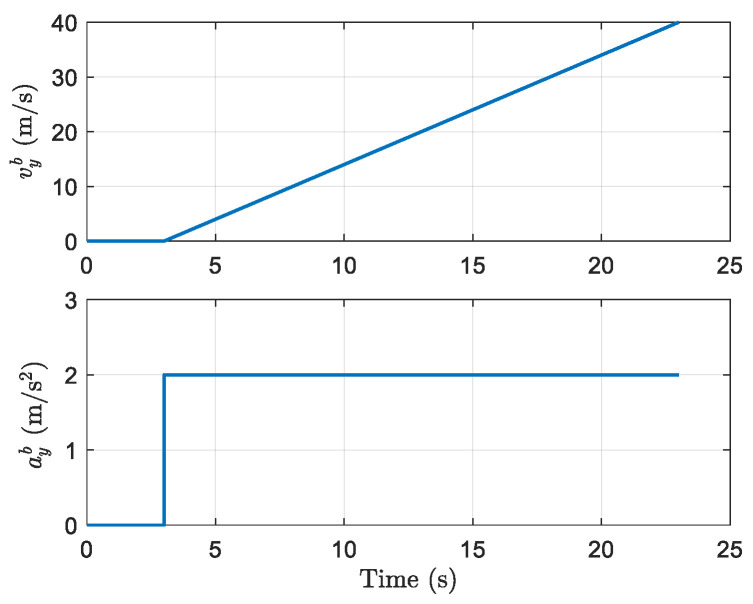
Forward velocity and acceleration profiles in the simulated trajectory.

The simulation assumes a runway heading of 20°, with the aircraft initially stationary at the runway starting point for 3 s. Subsequently, the aircraft accelerates at a rate of 2 m/s^2^ for 20 s, reaching a takeoff speed of 40 m/s. The total ground taxiing distance is 400 m with a total trajectory duration of 23 s. The simulated forward velocity and acceleration profiles are depicted in Figure 2.

To establish a realistic simulation environment, the inertial sensor parameters and initial navigation errors are summarized in Table 1. This setup explicitly characterizes both stochastic and deterministic error components, including bias repeatability, random walk, scale-factor error, and misalignment error for both gyroscopes and accelerometers, as well as the runway heading error. Additionally, the initial state uncertainties encompassing attitude, velocity, and position errors are incorporated to evaluate the system’s performance. These defined error bounds constitute the statistical foundation for the subsequent Monte Carlo simulations.

### 3.2. Covariance Analysis

Covariance analysis has been widely employed to evaluate the estimation performance and practical observability of integrated navigation systems, as it directly quantifies the reduction of estimation uncertainty. In this study, the degree of observability for the j-th state variable is defined as follows [36]:

(46) $$\sigma_{X_{k(j)}}=\sqrt{\frac{P_{0(j,j)}}{P_{k(j,j)}}}$$

where $X_{k}(j)$ denotes the j-th element of the error-state vector, $P_{0(j,j)}$ denotes the j-th diagonal element of the initial error covariance matrix, and $P_{k(j,j)}$ denotes the corresponding diagonal element of the posterior error covariance matrix at epoch k.

The degree of observability defined in Equation (46) is dimensionless and characterizes the reduction in estimation uncertainty for each individual state variable throughout the Kalman filtering process. A more pronounced reduction in the corresponding estimation error covariance implies enhanced observability. Conversely, a degree of observability close to unity indicates that the uncertainty of the corresponding state remains almost unchanged after filtering, implying limited practical observability.

For clarity, the practical observability of each state variable is categorized into four levels based on the following empirical criterion.

(47) $$\left\{\begin{array}{ll} \sigma_{X_{k(j)}}\leq 1, & \mathrm{Unobservable}\\ 1<\sigma_{X_{k(j)}}\leq 2, & \mathrm{Weakly}\;\mathrm{observable}\\ 2<\sigma_{X_{k(j)}}\leq 10, & \mathrm{Moderately}\;\mathrm{observable}\\ \sigma_{X_{k(j)}}>10, & \mathrm{Strongly}\;\mathrm{observable} \end{array}\right.$$

It is worth noting that the thresholds employed in Equation (47) are empirical criteria introduced to facilitate quantitative comparison among different error-state variables, rather than rigorous observability conditions.

**Figure 3:**
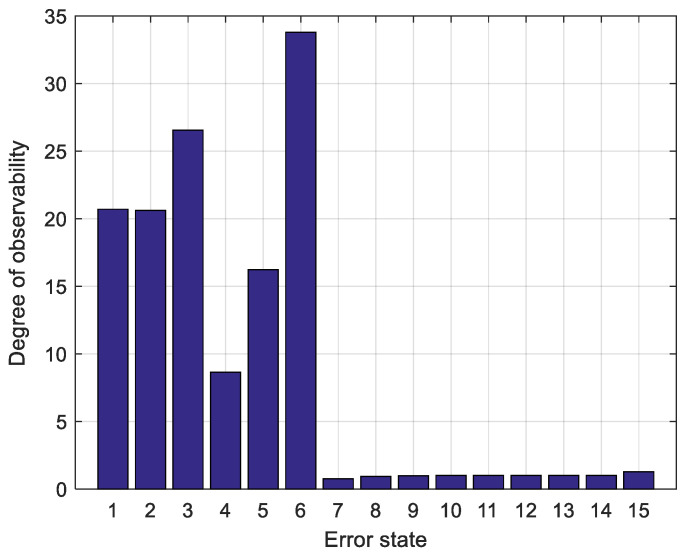
Practical observability indices obtained from covariance analysis.

Based on the trajectory design and filter parameter settings presented in Section 3.1, the covariance observability indices for the 15 error-state variables are shown in Figure 3, where the horizontal axis corresponds to the state index defined in Equation (4).

According to the observability criterion defined in Equation (47), the observability levels of all error-state variables are summarized in Table 2.

As illustrated in Figure 3 and Table 2, the horizontal misalignment angles (States 1–2), the azimuth misalignment angle (State 3), the forward velocity error (State 5), and the vertical velocity error (State 6) exhibit strong observability, with observability indices greater than 10. During the runway taxiing alignment process, their estimation uncertainties are substantially reduced, indicating that these states can be accurately estimated within a relatively short alignment interval.

The covariance analysis results indicate that the proposed runway-constrained alignment method significantly improves the estimation accuracy of attitude-related states and velocity error states, whereas position errors and most inertial sensor biases remain challenging to estimate accurately within the short runway taxiing interval.

### 3.3. Simulation Results

Since the proposed method estimates SINS misalignment angles by leveraging velocity and displacement constraints as measurements, velocity and position disturbances during runway taxiing constitute the primary factors affecting the alignment performance. In practical runway operations, the lateral velocity $v_{x}^{p}$ is predominantly influenced by crosswinds and sideslip, thereby exhibiting low-frequency characteristics. In contrast, the vertical velocity $v_{z}^{p}$ is primarily governed by runway unevenness and airframe vibration, giving rise to high-frequency disturbances. Based on the simulation conditions described in Section 3.1, sinusoidal functions are employed to model the lateral disturbance velocity $\Delta v_{xw}$ and the vertical disturbance velocity $\Delta v_{zw}$, which are expressed as follows:

(48) $$\left\{\begin{array}{l} \Delta v_{xw}=0.3\sin(0.5\pi t)\\ \Delta v_{zw}=0.1\sin(2\pi t) \end{array}\right.$$

**Figure 4:**
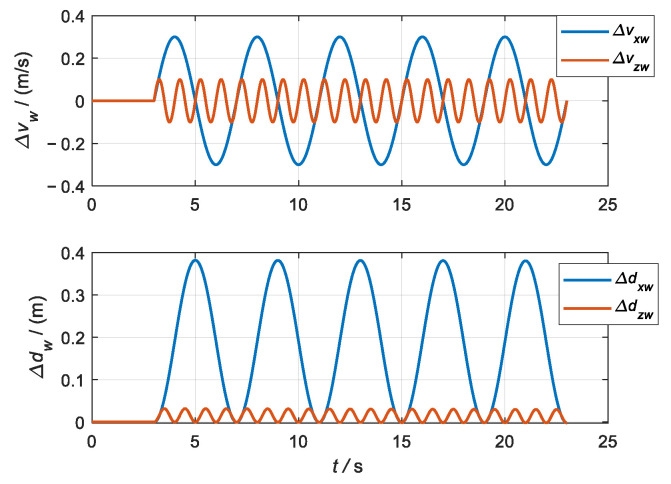
Simulated disturbance velocities and displacements.

The corresponding disturbance displacements are derived by integrating the disturbance velocities over time. The simulated disturbance velocities and the resulting disturbance displacements superimposed on the reference trajectory are illustrated in Figure 4.

In Figure 4, the lateral and vertical disturbance displacements, denoted by $\Delta d_{xw}$ and $\Delta d_{zw}$, are generated by integrating the corresponding disturbance velocities. It is evident that the disturbance displacement attains its maximum when the corresponding disturbance velocity is approximately zero, whereas the disturbance velocity peaks when the displacement varies most rapidly. Consequently, the velocity and displacement constraints provide complementary measurement information. When the disturbance velocity increases, the displacement constraint measurement accuracy is higher, whereas when the disturbance velocity approaches zero, the velocity measurement accuracy improves, thereby enhancing the overall measurement accuracy and strengthening the robustness of the proposed alignment method.

**Figure 5:**
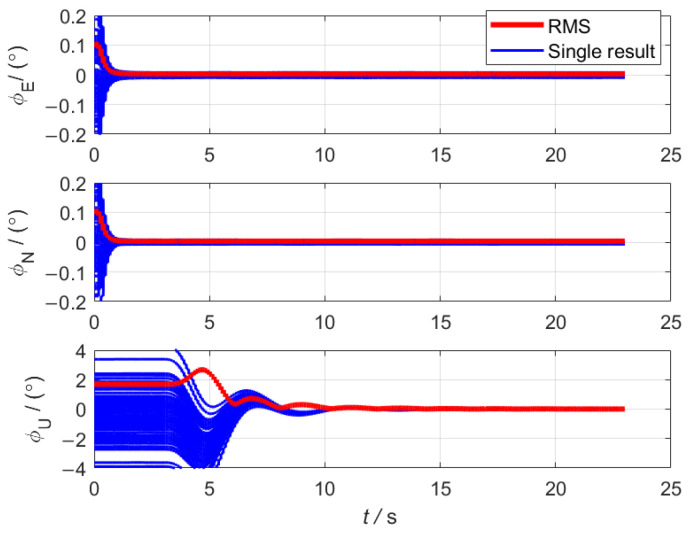
Misalignment angles estimated from 100 Monte Carlo simulations.

Based on the sensor error parameters specified in Table 1, 100 Monte Carlo simulations were conducted under the aforementioned disturbance conditions. The resulting misalignment angle estimation errors are depicted in Figure 5, which also illustrates the statistical performance (root-mean-square error, RMSE) of the proposed alignment method.

The statistical results of the alignment errors at the conclusion of the taxiing phase are summarized in Table 3.

As depicted in Figure 5 and Table 3, the horizontal misalignment angles $\phi_{E}$ and $\phi_{N}$ are rapidly estimated during the stationary phase. Upon the onset of taxiing motion at the third second, the azimuth misalignment angle $\phi_{U}$ converges rapidly owing to the excitation provided by the forward acceleration $a_{y}^{p}$. According to the theoretical accuracy limits established in [11], the stationary base azimuth alignment accuracy for the sensor grades specified in Table 1 is approximately 0.1°. However, under the excitation of accelerated motion, the proposed method achieves a final estimation accuracy of 0.0087° for the azimuth misalignment angle, as shown in Table 3, thereby showing improved accuracy relative to the stated stationary-alignment limit.

As shown in Table 3, owing to the brief alignment duration, the velocity and position errors remain small at the conclusion of the alignment process. Furthermore, the proposed constrained alignment method is unable to effectively estimate the gyroscope biases and horizontal accelerometer biases; nevertheless, it exhibits a moderate estimation capability for the vertical accelerometer bias, achieving a final accuracy of approximately 32 μg. The simulation results are in agreement with the covariance analysis findings.

**Figure 6:**
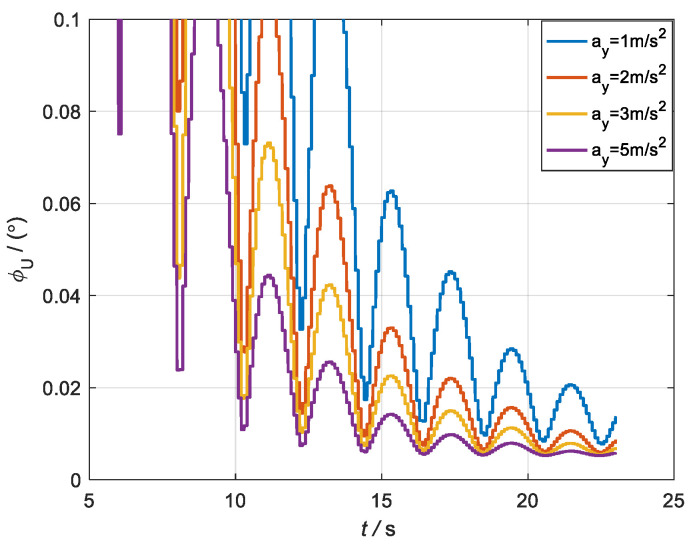
RMS azimuth misalignment errors under varying acceleration profiles.

During runway taxiing, the aircraft longitudinal acceleration serves as a critical factor in enhancing the observability of the azimuth misalignment angle. Based on the nominal simulation trajectory described in Section 3.1, four constant acceleration profiles of 1, 2, 3, and 5 m/s^2^ are considered. For each profile, 100 Monte Carlo simulations are conducted using the sensor error parameters specified in Table 1. The root-mean-square azimuth alignment errors under the aforementioned acceleration profiles are shown in Figure 6.

In Figure 6, the fluctuations in the azimuth misalignment angle arise from the combined effects of the disturbance velocities depicted in Figure 4 and the simulated sensor errors. It is evident that increasing the acceleration effectively attenuates the oscillation of the azimuth misalignment angle and substantially enhances the alignment accuracy. When the acceleration reaches 5 m/s^2^, the azimuth alignment error converges most rapidly and achieves the highest alignment accuracy, which corroborates the findings of the covariance analysis.

## 4. Flight Validation

Flight tests were conducted using a Cessna 172 aircraft manufactured by Cessna Aircraft Company (Wichita, KS, USA), which features a maximum speed of 228 km/h, a maximum takeoff weight of 1113 kg, and a service ceiling of 4116 m.

**Figure 7:**
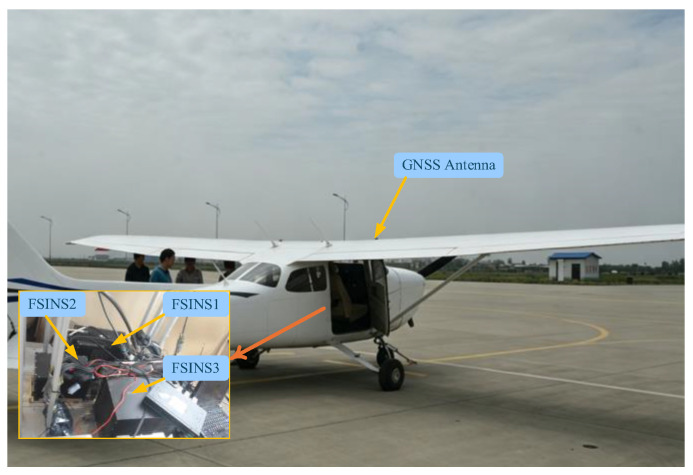
Test aircraft and experimental setup.

Three sets of fiber optic gyroscope SINS (FSINS) were installed for the taxiing alignment experiments, designated as FSINS1, FSINS2, and FSINS3. The three systems exhibit nearly identical performance specifications, with a gyroscope bias repeatability of 0.02°/h and an accelerometer bias of 50 μg. The detailed device specifications correspond to those listed in Table 1. Each of the three systems was equipped with an embedded GNSS receiver, sharing a common GNSS antenna via a power splitter. The test aircraft and experimental setup are depicted in Figure 7.

**Figure 8:**
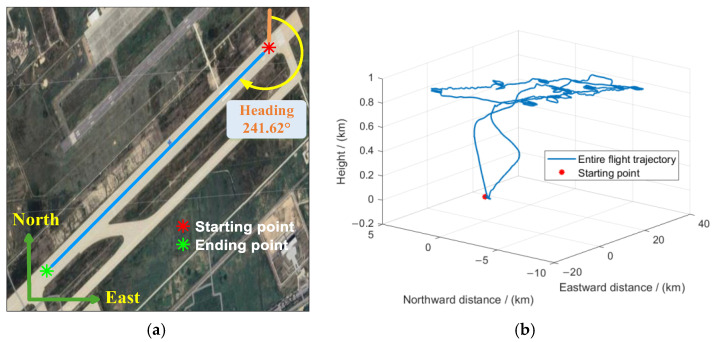
Flight trajectory. (**a**) Actual taxiing path overlaid on satellite imagery of the runway; (**b**) Complete 1.6 h flight profile.

The actual flight trajectory is depicted in Figure 8. Figure 8a presents the actual taxiing trajectory superimposed on the satellite map, with a precisely measured runway heading of 241.62°. Figure 8b illustrates the entire 1.6 h flight profile, which contains sufficient intentional maneuvers to enable the derivation of a high-precision heading reference benchmark through post-processing.

Establishing a highly accurate heading reference in a dynamic environment presents a formidable challenge. To overcome this limitation, a dedicated post-processing methodology is employed in this study. The recorded raw sensor data are first processed using an INS/post-processed differential GNSS integrated navigation framework, and the obtained navigation solution is subsequently smoothed using the Rauch–Tung–Striebel (RTS) smoothing algorithm. The large-amplitude intentional maneuvers incorporated throughout the entire flight sequence, as illustrated in Figure 8b, effectively compensate for the observability limitations in the runway taxiing phase, thereby enabling post-processing methods to generate high-precision attitude, velocity, and position benchmarks for this phase. Based on prior experimental evaluations, the post-processing method attains centimeter-level position accuracy and dynamic heading accuracy better than 0.01°.

**Figure 9:**
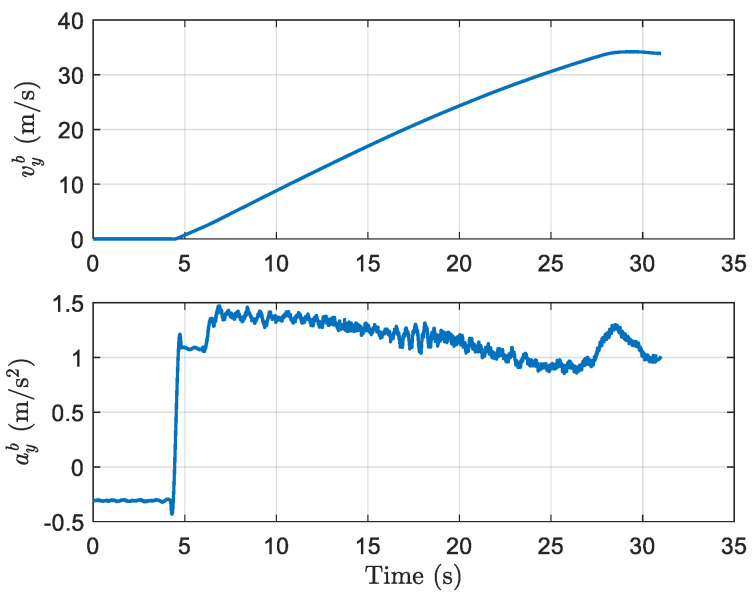
Forward velocity and acceleration profiles.

The forward velocity and acceleration profiles throughout the taxiing phase are depicted in Figure 9.

As shown in Figure 9, the aircraft remains stationary at the runway starting point for 3.5 s, undergoes a 24 s taxiing acceleration phase, and initiates the climb segment at 27.5 s. The longitudinal acceleration during the ground roll is approximately 1 m/s^2^, the takeoff speed reaches 34.2 m/s, and the total runway taxi distance is approximately 410 m.

Four processing schemes were employed to handle the taxiing alignment data for each FSINS:

Scheme 1: In-motion alignment aided by GNSS single-point positioning data, based on the algorithm detailed in [1].

Scheme 2: The proposed runway-heading-aided in-motion alignment algorithm, utilizing solely the velocity-constrained measurement model (27).

Scheme 3: The proposed runway-heading-aided in-motion alignment algorithm, utilizing solely the displacement-constrained measurement model (35).

Scheme 4: The proposed runway-heading-aided in-motion alignment algorithm, integrating both the velocity-constrained measurement model (27) and the displacement-constrained measurement model (35).

**Figure 10:**
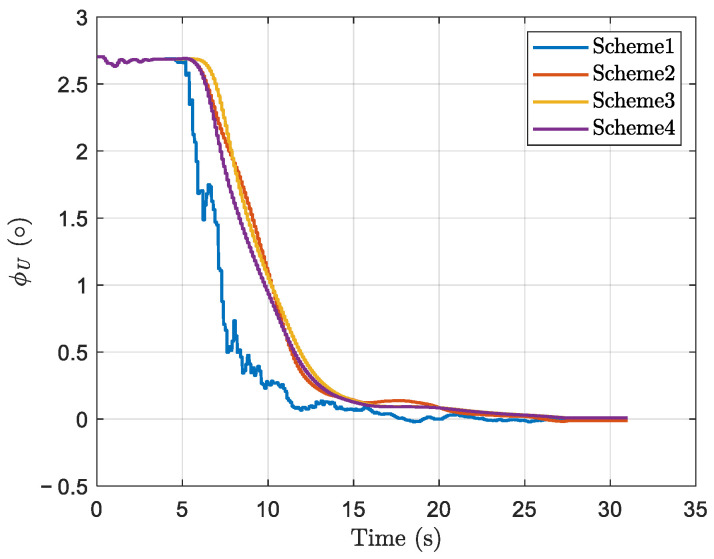
Azimuth alignment errors of FSINS1.

**Figure 11:**
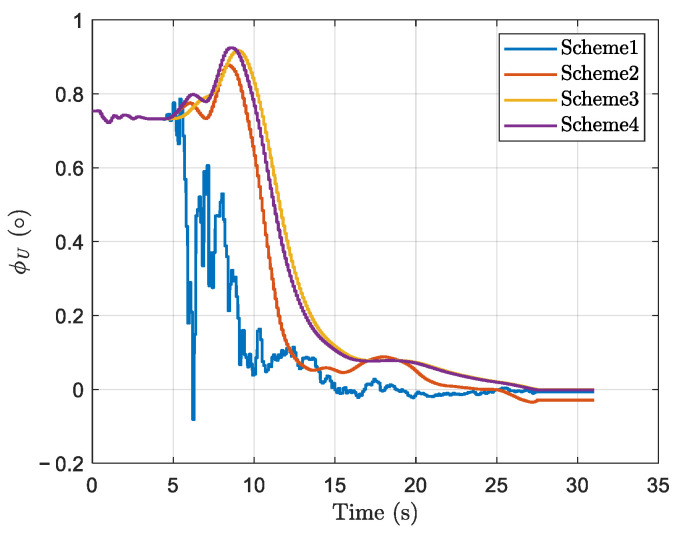
Azimuth alignment errors of FSINS2.

**Figure 12:**
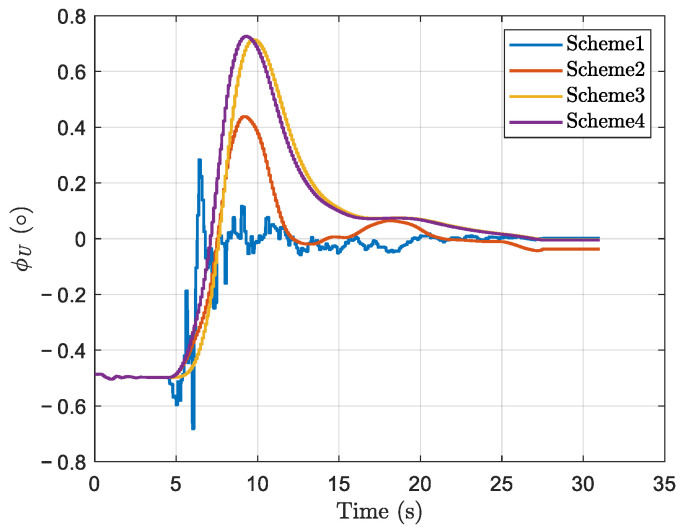
Azimuth alignment errors of FSINS3.

The azimuth alignment errors corresponding to different methods and FSINS units are presented in Figure 10, Figure 11 and Figure 12.

As shown in Figure 10, Figure 11 and Figure 12, owing to the varying mounting misalignments of the three FSINS units, the initial azimuth errors after runway heading assignment are 2.7°, 0.75°, and −0.49°, respectively. During the aircraft’s acceleration phase, the azimuth misalignment angles for all four methods converge rapidly toward the reference values. Scheme 1, which is aided by GNSS velocity and position, adopts full three-dimensional position and velocity measurements and thus exhibits the fastest convergence. However, it suffers from the most prominent fluctuations, which are induced by the GNSS single-point positioning errors. In contrast, the other three runway-heading-aided schemes (Schemes 2–4) show a slightly lower convergence rate yet complete the in-motion alignment without relying on external aiding equipment, delivering smooth convergence curves. Specifically, the velocity-constrained Scheme 2 converges quickly but retains a certain steady-state error from velocity interference; the displacement-constrained Scheme 3 achieves higher accuracy but converges more slowly; and the dual-constrained Scheme 4 integrates both advantages, simultaneously attaining high alignment accuracy and rapid convergence.

**Figure 13:**
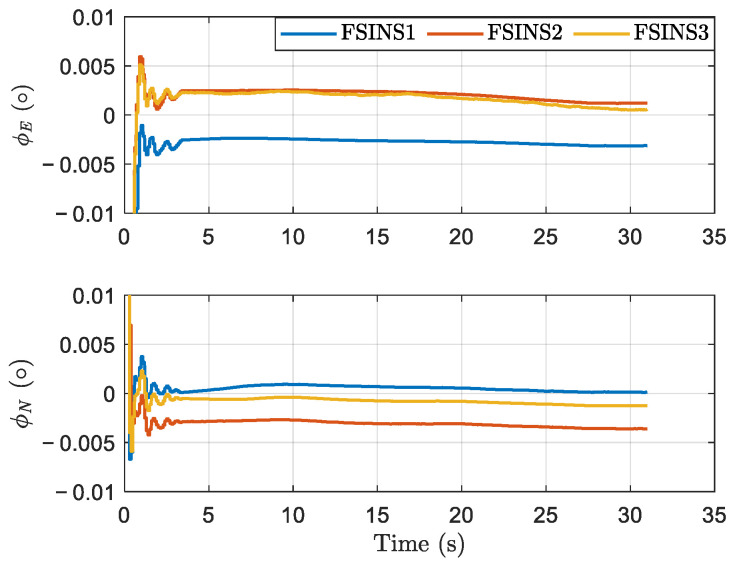
Horizontal misalignment angles of Scheme 4.

**Figure 14:**
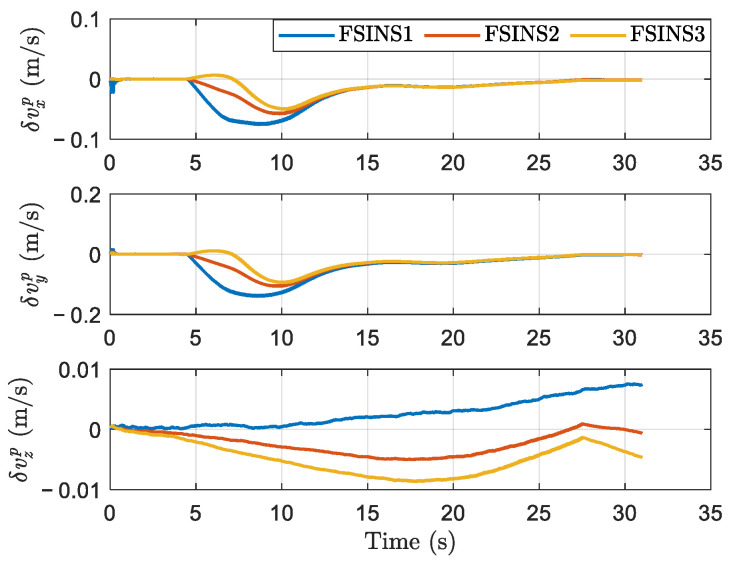
Velocity errors of Scheme 4.

**Figure 15:**
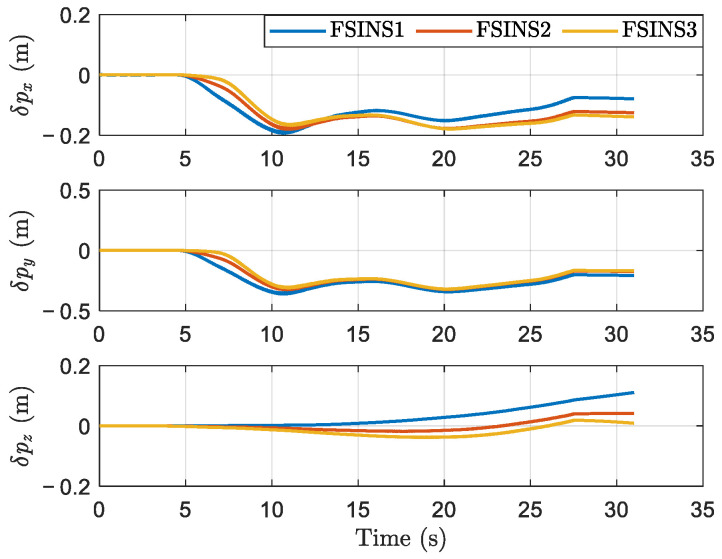
Position errors of Scheme 4.

For brevity, except for the azimuth misalignment angle, only the horizontal misalignment, velocity error, and position error curves of Scheme 4 are presented in Figure 13, Figure 14 and Figure 15.

As seen in Figure 13, Figure 14 and Figure 15, the horizontal misalignment angles converge rapidly during the initial 3 s stationary period, and the velocity and position errors remain minimal throughout the alignment process.

At the end of the taxiing phase, the final alignment error statistics for the three FSINS units are summarized in Table 4.

Table 4 demonstrates that the proposed runway-heading-aided alignment algorithms (Schemes 2–4) can all complete rapid alignment within 30 s during the taxiing phase, with both alignment time and accuracy exceeding traditional stationary self-alignment methods. Without relying on GNSS or external equipment, Schemes 3 and 4 attain an azimuth alignment accuracy of approximately 0.005–0.006°, which is comparable to that of Scheme 1 (GNSS-aided).

## 5. Conclusions

This paper proposes a rapid self-alignment method for fixed-wing aircraft during the taxi-to-takeoff phase. The estimation performance of the proposed method on various error states is revealed through covariance analysis. Finally, the effectiveness of the method is validated via both numerical simulations and flight experiments. The main conclusions are as follows:

(1) The taxiing self-alignment method utilizes the acceleration during runway taxiing to facilitate error estimation. The proposed method simultaneously improves the convergence speed and accuracy of the initial alignment, while ensuring accurate velocity estimation and stable position performance at the completion of the alignment phase.
(2) The proposed method is governed by runway heading constraints and specific kinematic models. Consequently, it is not applicable to rotary-wing aircraft.
(3) The taxiing self-alignment method requires no external aiding sensors and relies solely on the pre-surveyed runway heading data, thereby ensuring both high autonomy and practical engineering applicability.

This method can serve as an emergency alignment approach for the INS of fixed-wing aircraft. Furthermore, the method may be extended to autonomous alignment in polar regions and to taxiing-to-takeoff alignment for carrier-based aircraft, thereby enhancing the aircraft’s emergency response capability.

## Figures and Tables

**Table 1:** Simulation error parameters.

Category	Parameter	Value
Gyroscopes	Bias repeatability (1σ)	0.02°/h
	Angle random walk	0.002°/√h
	Scale-factor error (1σ)	30 ppm
	Misalignment error (1σ)	3 arcsec
Accelerometers	Bias repeatability (1σ)	50 μg
	Velocity random walk	10 μg/√Hz
	Scale-factor error (1σ)	50 ppm
	Misalignment error (1σ)	3 arcsec
Runway	Runway heading error (1σ)	0.005°
Initial errors	Attitude error (1σ)	[0.1; 0.1; 1.5]°
	Velocity error (1σ)	[0.1; 0.1; 0.1] m/s
	Position error (1σ)	[0.5; 0.5; 0.5] m

**Table 2:** Covariance-based observability evaluation of the 15 error-state variables.

Error Index	Error State	Degree of Observability	Classification
1–2	$\phi_{E}$, $\phi_{N}$	20.7032, 20.6136	Strong
3	$\phi_{U}$	26.5420	Strong
4	$\delta v_{x}^{p}$	8.6454	Medium
5	$\delta v_{y}^{p}$	16.2384	Strong
6	$\delta v_{z}^{p}$	33.7952	Strong
7–9	δp	0.7660, 0.9333, 0.9825	Unobservable
10–12	$\varepsilon^{b}$	1.0000, 1.0000, 1.0000	Unobservable
13–14	$\nabla_{x}^{b}$, $\nabla_{y}^{b}$	1.0004, 1.0018	Weak
15	$\nabla_{z}^{b}$	1.2747	Weak

**Table 3:** Final alignment result statistics (RMSE).

Error Type	Value (RMSE)
Attitude Misalignment (°)	[0.0029; 0.0033; 0.0087]
Velocity Error (m/s)	[0.0051; 0.0058; 0.0051]
Position Error (m)	[0.1693; 0.4879; 0.0373]
Gyroscope Bias Estimation Error (°/h)	[0.0184; 0.0205; 0.0169]
Accelerometer Bias Estimation Error (μg)	[52.4260; 48.6688; 32.1705]

**Table 4:** Final alignment results.

Scheme	Horizontal Misalignment (°)	Azimuth Misalignment (°)	Velocity Error (m/s)	Position Error (m)
Scheme 1	0.0025	0.0053	0.0079	0.1101
Scheme 2	0.0022	0.0282	0.0121	0.1832
Scheme 3	0.0021	0.0056	0.0033	0.1362
Scheme 4	0.0021	0.0054	0.0032	0.1324

## Data Availability

The datasets generated and analyzed during this study are not publicly available due to confidentiality requirements and institutional restrictions related to the flight test data. Requests for access to the data may be directed to the corresponding authors and will be considered on a reasonable basis, subject to permission from the data owner.

## Abbreviations

The following abbreviations are used in this manuscript:

INS	Inertial Navigation System
SINS	Strapdown Inertial Navigation System
IFA	In-Flight Alignment
GNSS	Global Navigation Satellite System
CNS	Celestial Navigation System
LDV	Laser Doppler Velocimetry
IMU	Inertial Measurement Unit
RTS	Rauch–Tung–Striebel
KF	Kalman Filter
NHC	Non-Holonomic Constraint
ARW	Angular Random Walk
PSD	Power Spectral Density
VRW	Velocity Random Walk
RMS	Root Mean Square
RMSE	Root Mean Square Error
RFU	Right–Forward–Up
ENU	East–North–Up
ZUPT	Zero-Velocity Update
FSINS	Fiber optic gyroscope SINS

## Appendix A

### Initialization of the Kalman Filter

The initial state vector, initial error covariance matrix, process-noise covariance matrix, and measurement-noise covariance matrix of the proposed Kalman filter are initialized as follows:

(A1) $$X_{0}=0_{15\times 1}$$

where the initial attitude, velocity, and position errors, along with the gyroscope and accelerometer biases, are assumed to be zero.

The initial error covariance matrix is defined as follows:

(A2) $$P_{0}=\mathrm{diag}{\left(\left[\sigma_{\phi };\;\sigma_{v};\;\sigma_{p};\;\sigma_{\varepsilon };\;\sigma_{\nabla }\right]\right)}^{2}$$

where $\sigma_{\phi }$ = [0.1; 0.1; 1.5]°, $\sigma_{v}$ = [0.3; 0.3; 0.3] m/s, $\sigma_{p}$ = [0.5; 0.5; 0.5] m, $\sigma_{\varepsilon }$ = [0.05; 0.05; 0.05]°/h, $\sigma_{\nabla }$ = [50; 50; 50] μg.

The PSD matrix for continuous-time process noise is initialized as follows:

(A3) $$Q_{t}=\left[\begin{array}{ccc} q_{g}I_{3} & 0_{3\times 3} & 0_{3\times 6}\\ 0_{3\times 3} & q_{a}I_{3} & 0_{3\times 6}\\ 0_{6\times 3} & 0_{6\times 3} & 0_{6\times 6} \end{array}\right]$$

where the PSDs of the gyroscope and accelerometer noise processes are set to $q_{g}={\left(0.002{}^{\circ}/\sqrt{h}\right)}^{2}$ and $q_{a}={\left(10\;\mu g/\sqrt{\mathrm{Hz}}\right)}^{2}$, respectively. The corresponding discrete-time process-noise covariance matrix is obtained using (25).

During the stationary alignment stage, the measurement-noise covariance matrix associated with the zero-velocity constraint is set as follows:

(A4) $$R_{v0}=\mathrm{diag}{\left(\left[0.003;\;0.003;\;0.003\right]\;m/s\right)}^{2}$$

During the taxiing phase, the measurement-noise covariance matrices for the velocity and displacement constraints are configured as follows:

(A5) $$R_{v}=\mathrm{diag}{\left(\left[0.3;\;0.3\right]\;m/s\right)}^{2}$$

(A6) $$R_{s}=\mathrm{diag}{\left(\left[0.5;\;0.5\right]\;m\right)}^{2}$$
